# Atenolol in the prophylaxis of chronic migraine: a 3-month open-label study

**DOI:** 10.1186/2193-1801-2-479

**Published:** 2013-09-22

**Authors:** Bengt Edvardsson

**Affiliations:** Department of Neurology, Faculty of Medicine, Skane University Hospital, S-221 85 Lund, Sweden

**Keywords:** Headache, Migraine, Chronic migraine, Chronic daily headache, Beta blockers, Atenolol

## Abstract

**Background:**

Chronic migraine (CM) is a type of chronic daily headache. CM presents a challenge to primary care physicians and neurologists. Any new treatment showing efficiency would therefore be of great importance. Atenolol together with other beta-blockers is a first-line choice in episodic migraine prophylaxis. Clinical findings support the efficacy of atenolol in doses of 50 to 200 mg/day.

**Methods:**

Here I present an open-label study the aim of which is to evaluate the efficacy and tolerability of atenolol (50 mg o.d) for the prevention of CM. 19 patients affected by CM were studied.

**Results:**

Following a one-month run-in period, the patients took atenolol for 3 months. Mean numbers of headache days per month were reduced from 20.1 ± 2.4 during the run-in period to 7.8 ± 6.1 by month 1.5 and to 7.1 ± 5.7 by the 3rd month of treatment (p < 0.0003). There was a significant difference between 1.5 months and the 3rd month (p < 0.006). The severity of attacks was reduced from a mean 2.3 ± 0.6 to 1.4 ± 1.1 (p < 0.010) at 1.5 months. In this, there was no difference between 1.5 months and the 3rd month. In 5 (29%) of the17 patients who completed the study, CM was totally gone during the 3rd month of treatment. No patient was totally unresponsive to the drug. Among the patients who completed the study, the treatment was well tolerated and the compliance was good.

**Conclusion:**

Atenolol seems to be a safe and effective treatment for CM. Controlled trials are needed to confirm the observed results.

## Introduction

Chronic migraine (CM) is a type of chronic daily headache. CM presents a challenge to primary care physicians and neurologists. The overall cost of migraine to society is large (Hu et al. 1999). Any new treatment showing efficiency is thus of great importance.

CM was a new addition to the revised International Classification of Headache Disorders (ICHD) criteria published in 2004 (Headache Subcommittee of the International Headache Society 2004). The criteria for CM were revised in 2006 and are included in the appendix of the current classification (Headache Classification Committee, Olesen et al. 2006). CM patients often require multiple drugs and nondrug treatment modalities. The pathophysiology of CM is not clear; however the basic underlying pathophysiology is the disposition to migraine without aura. The prognosis and treatment of patients with CM are variable.

Most preventive agents used for CM have not been examined in well designed double-blind studies (Olesen et al. 2006). Atenolol is together with other beta-blockers a first-line choice in episodic migraine prophylaxis. Clinical findings support the efficacy of atenolol in doses of 50 to 200 mg/day (Olesen et al. 2006). Results from three trials confirm the benefit of atenolol in episodic migraine prophylaxis. (Stensrud et al. 1980) in their study found no statistically significant difference between atenolol 50 mg b.i.d. and propranolol 80 mg b.i.d. Atenolol was more effective than placebo. Another study (Forssman et al. 1983) reported that the effect of atenolol 100 mg o.d. was significantly better than that of placebo. Interestingly, the intake of ergotamine products was significantly lower in all patients using such drugs. (Johannsson et al. 1987) also confirmed that the effect of atenolol 100 mg o.d. is significantly better than that of placebo. Few side effects were reported with both atenolol and placebo. The study showed atenolol to be safe and effective in the prophylactic treatment of episodic migraine.

The aim of this open-label study was to evaluate the possible efficacy and tolerability of atenolol in the prophylaxis of CM.

## Material and methods

For this open-label prospective study a sample of 19 patients were enrolled, aged 19–32 years (5 M, 14 F). All patients satisfied the criteria for CM (Headache Classification Committee, Olesen et al. 2006). Patients having the diagnosis CM with medication overuse were excluded. Patients were consecutively recruited from the Outpatient department of the Department of Neurology at Skane University Hospital, Lund.

Inclusion criteria were: initial onset of CM at least 1 year before and patients over 18 years of age. Exclusion criteria were: other headaches but migraine, other forms of chronic pain, overuse of pain/migraine medication, psychiatric diseases, neurological diseases and other chronic diseases, intake of CNS-active medications the last 3 months before the selection, intake of prophylactic medication for migraine the last 3 months before the selection, intake of other regular medications, pregnancy or risk of pregnancy and inability/unwillingness to cooperate. An informed consent was obtained from each participant. Characteristics of patients enrolled are displayed in Table 1.

**Table 1:** **Characteristics of the population studied**

Sex	
Male	5
Female	14
Age (years)	
Mean	25.1
Median	25
Range	19-32
Disease (years)	
Mean	3,1
Median	3

After a one-month run-in period, patients received atenolol as treatment in a low dose (50 mg o.d.) for 3 months. The inclusion criteria were re-examined at the end of the run-in period. The patients were free to take symptomatic medication, if needed. Headache days per month and severity (rated as: 1, mild; 2, moderate; and 3, severe) of migraine attacks were recorded by the patients in a headache diary. Headache days per month were the main outcome measure and the principal index used to evaluate the efficacy. Headache days and severity during the run-in period was compared with what was found after 1.5 months and during the 3rd month of therapy. The Wilcoxon signed rank test (a nonparametric test) was used for the statistical analysis. Differences were considered significant when P value was less than 0.05. Adverse events were reported during the period.

## Results

**Figure 1:**
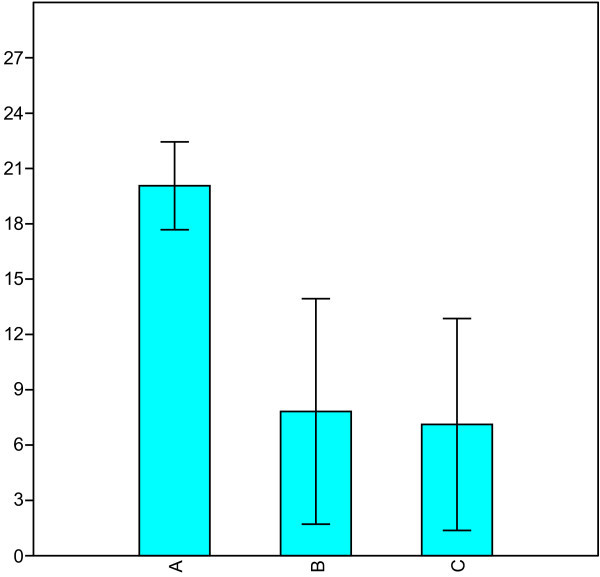
**Mean (± SD) headache days per month. (A)** and at 1.5 months **(B)** and month 3 **(C)**.

**Figure 2:**
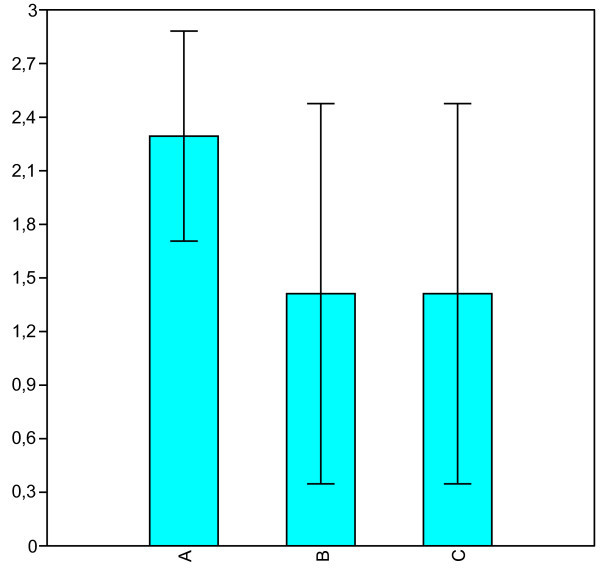
**Mean (± SD) severity of migraine attacks during the study.(A)** and at 1.5 months **(B)** and month 3 **(C)**.

Two patients out of 19 enrolled left the study because of adverse events: fatigue and dizziness. 17 patients completed the treatment period of 3 months. Mean headache days per month were reduced from 20.1 ± 2.4 during the run-in period to 7.8 ± 6.1 at 1.5 months and to 7.1 ± 5.7 by the 3rd month of treatment (p < 0.0003). There was a significant difference between 1.5 months and the 3rd month (p < 0.006) (Figure 1). The severity of attacks was reduced from mean 2.3 ± 0.6 to 1.4 ± 1.1 (p < 0.010) at 1.5 months. There were no difference between 1.5 months and the 3rd month (Figure 2). In 5 (29%) of the17 patients who completed the study, CM was totally gone at the 3rd month of treatment. No patient was totally unresponsive to the drug. No significant adverse effects were reported among the patients who completed the study. Baseline systolic blood pressure and heart rate did not differ considerably during the period. The medication had no effects on activities of daily living and the patients reported improved quality of life due to relief of symptoms.

## Discussion

This is an open label study made to evaluate the possible efficacy and tolerability of atenolol in CM. The results in the study indicate benefit in preventing CM by significantly reducing the number of headache days per month at 1.5 months and in the 3rd month of treatment compared with the run-in period. Atenolol was also able to significantly reduce the severity of the attacks at 1.5 months and in the 3rd month of treatment compared with the run-in period. To my knowledge, this is the first prospective study of atenolol as preventive treatment for CM.

The study contradicts earlier results in CM. No beta-blocker has a Class I study showing effectiveness in reducing CM (Couch 2011). Up to know, only topiramate and local injections of botulinum toxin have shown efficacy in large placebo-controlled randomized trials (Couch 2011).

However, these results are in accordance with previous studies including migraineurs in whom an effect of prophylactic atenolol has been shown. Beta-blockers are approximately 50% effective in producing a > 50% reduction in attack frequency (Stensrud & Sjaastad 1980; Forssman et al. 1983; Johannsson et al. 1987; Olesen et al. 2006). Propranolol is effective in migraine prevention at a daily dose of 80–240 mg (Barbanti et al. 2011). A Cochrane review of studies 2004 concluded that propranolol is effective in preventing migraine in the short term (Barbanti et al. 2011). The relative efficacy of the different beta-blockers has not been established. Most studies show no significant difference between drugs.

The action of beta-blockers is most likely central. Blockade of β1-mediated effects and consequent inhibition of Na^+^ release and tyrosine hydroxylase activity are considered the main mechanisms of action. Beta-blockers reduce the neuronal firing rate of noradrenergic neurons of the locus coeruleus, regulate the firing rate of PAG neurons and probably interact with the serotonergic system by blocking 5-HT2C and 5-HT2B receptors. It has been hypothesized that beta-blockers exert some of their prophylactic effects in migraine through an action at the ventroposteromedial thalamic nucleus and inhibition of cortical spreading depression (Barbanti et al. 2011).

Earlier studies have shown that compared with episodic migraine, patients suffering from CM are more likely to be depressed, anxious, suffering from other forms of chronic pain, and overusing acute pain medications. Epidemiologic and clinical research consistently documents an association between depressive, bipolar, and anxiety disorders with migraine (Diener et al. 2011; Olesen et al. 2006). All beta-blockers can cause behavioural adverse events as fatigue, lethargy and depression (Nappi & Moskowitz 2011). Because propranolol may predispose to depression, its use as an antimigraine preventive agent is limited (Couch 2011). However, in this study the subjects showed no signs of depression. The subjects were young and otherwise healthy. No medication overuse was found. These facts may have contributed to the good efficacy results.

Atenolol is associated with risks for pregnant and lactating women as well as for diabetics. Studies have shown that women with chronic hypertension that is treated with atenolol have higher rates of intrauterine growth restriction and preterm delivery (Orbach et al. 2013). Atenolol is also associated with significant effects on some nursing infants and should be given to nursing mothers with caution. There is one report of hypotension, bradycardia, and cyanosis in a breast-fed infant of a mother taking 100 mg daily (Hutchinson et al. 2013). Beta- blockers are also contraindicated in patients with brittle diabetes mellitus (Olesen et al. 2006).

Although the patients were observed prospectively, the study has limitations. It is limited by its small sample size and open-label nature. The good efficacy results obtained here must be interpreted with caution, as they come from an open research in a condition with a high placebo response. Nevertheless, the patients were carefully selected and all patients satisfied the criteria for CM. In the study, the persistence of therapeutic effect (29% of patients being headache-free since the run-in period) can hardly be attributed to a placebo effect only.

## Conclusion

Atenolol seems to be a safe and effective treatment for CM. Further investigations with double-blind, placebo-controlled randomized trials would be of value in order to assess the real efficacy of atenolol as a new therapeutic option for preventing CM.

## Consent

Written informed consent was obtained from the patient for the publication of this report and any accompanying images.

## Abbreviations

PAG: Pontomesencephalic dorsolateral periaqueductal gray matter
5-HT2C: 5-hydroxytryptamine, 5-HT2C), Serotonin (5-HT) 2C receptors
5-HT2B: 5-hydroxytryptamine, 5-HT2B), Serotonin (5-HT) 2B receptors.
